# The impact of Er:YAG laser combined with fluoride treatment on the supragingival plaque microbiome in children with multiple caries: a dynamic study

**DOI:** 10.1186/s12903-022-02537-z

**Published:** 2022-11-24

**Authors:** Huang Wenyan, Zheng Pujue, Huang Yuhang, Liu Zhenni, Wu Yuejun, Wu Wenbin, Li Ziling, Janak L. Pathak, Zeng Sujuan

**Affiliations:** Department of Pediatric Dentistry, Affiliated Stomatology Hospital of Guangzhou Medical University, Guangdong Engineering Research Center of Oral Restoration and Reconstruction, Guangzhou Key Laboratory of Basic and Applied Research of Oral Regenerative Medicine, Guangzhou, 510182 Guangdong China

**Keywords:** Er:YAG laser, Fluoride, Caries prevention, Microbial diversity, Ecological balance

## Abstract

**Background:**

As a minimally invasive tool for caries prevention tool, the pulsed erbium:yttrium-aluminum-garnet (Er:YAG) laser is being used in a large number of studies. Microorganisms are extremely vital in the occurrence and development of dental caries. However, the impact of Er:YAG laser irradiation combined with fluoride on the dynamic microbial changes that occur in dental plaques is still uncertain. In this study, we examined the effect of an Er:YAG laser combined with fluorine on supragingival microbial composition and diversity in children with multiple caries.

**Methods:**

In this study, dental plaque samples (*n* = 48) were collected from 12 children with over 8 filled teeth. Supragingival plaques from left mandibular molars before (CB) and after fluoride treatment (CA) and right mandibular molars before (EB) and after fluoride+Er:YAG laser treatment (EA) were collected from each patient. In CB and EB groups, the samples were collected just before the treatments. In CA and EA groups, the samples were collected 1 month after treatments. Then, all specimens were subjected to 16S rRNA high-throughput sequencing to investigate the changes in microbial composition and diversity in mandibular molar supragingival plaques before and after fluoride or fluoride+Er:YAG laser treatment.

**Results:**

The dental plaque microbial diversity was higher in the EA group than in the EB group (baseline levels), and the microbial composition changed in EA group compared with EB group (*P* < 0.05). The levels of microorganisms associated with caries occurrence, including Proteobacteria, Fusobacteria, and Bacteroidetes, declined, while the levels of Faecacterium, Fastidiosipila, Vibrio, and Shewanella increased in EA group compared with EB group. The declines in Firmicutes, Streptococcus, Fusobacterium, and Veillonella levels were significantly lower in the EA group than in the CA group.

**Conclusion:**

The combined application of the Er:YAG laser and fluoride may be more effective than using fluoride alone in reducing the proportion of cariogenic bacteria, increasing the diversity of plaque microorganisms, and further promoting the microecological balance.

**Supplementary Information:**

The online version contains supplementary material available at 10.1186/s12903-022-02537-z.

## Background

As a considerably common and frequently occurring disease around the world, caries is a chronic, progressive and destructive disease of dental hard tissue caused by cariogenic bacteria, which can potentially lead to tooth loss and negatively affect human health [1, 2]. According to the results of the fourth Chinese oral epidemiological survey, the prevalence rate of caries among 5-year-old children was found to be 71.9%, which was 5.8% higher than that observed 10 years prior [3]. Despite the variety of public health caries prevention measures adopted in China over the last few years, the incidence of caries in children has continuously increased, and there are extensive challenges regarding the prevention and treatment of caries. Among all traditional caries prevention measures, the use of fluoride is the most extensively adopted clinical method, and it can reduce the demineralization of enamel, promote remineralization, interfere with the adhesion of bacterial plaque, and inhibit bacterial acid production [4–6]. However, the safety of fluoride use remains a controversial issue, since high doses of fluoride intake can negatively affect human health [7]. In this context, determining a method to improve the efficiency of caries prevention while reducing the fluoride dose is urgent in pediatric dentistry.

In 1997, the erbium:yttrium-aluminum-garnet (Er:YAG) laser was approved by the American Food and Drug Administration (FDA) for the treatment of dental hard tissue in humans [8, 9]. With the advantages of being safe, comfortable, minimally invasive, and free of high-frequency vibration, the Er:YAG laser is widely used in the treatment of oral diseases [10]. In the field of caries prevention, the Er:YAG laser can reduce the number of cariogenic microorganisms and enhance the resistance of enamel to acid erosion by melting by changing the crystallinity and reducing enamel permeability [11, 12]. However, the interaction between the laser and hard tissue is affected by the irradiation parameters, such as pulse duration, energy, and frequency [13, 14], and the appropriate irradiation parameters for caries prevention have not been established. Pagano et al. [15] analyzed clinical studies of the Er:YAG laser used alone or in combination with other traditional caries prevention measures in the past 10 years. In all of the studies, subablation parameters were used, ranging from 10 to 85 J/cm^2^. Several researchers have also expressed the belief that enamel surface heating induced by subablation parameters was less than 300 °C, which is below the ablation threshold and limits the damage to the tooth surface [4].

As shown in previous studies, the combination of Er:YAG laser and fluoride can change the chemical composition of tooth enamel, thereby promoting remineralization [16]. Through an in vitro experiment, Assarzadeh et al. [17] pointed out that the combination of fluoride and Er:YAG laser irradiation promoted the formation of a “fluoride reservoir” in tooth tissue and further enhanced the absorption of fluoride. This combination exhibited synergistic effects that were more pronounced than when each method was used alone. After a retrospective study of 118 studies in the 10 years from 2008 to 2018, Ma et al. [18] highlighted that the Er:YAG laser combined with fluoride had a more lasting effect on caries prevention, but such a combination has not been routinely used in clinical practice, and only a small number of researchers have conducted clinical experiments. An imbalance in microbial flora contributes to the occurrence and development of dental caries [19]. Bernardo et al. found that an increase in the surface roughness of permanent premolar teeth caused by Er:YAG laser irradiation (12.7, 25.5, and 38.2 J/cm^2^) did not affect *Streptococcus mutans* and *S. sanguinis* adhesion [20], while the use of an energy density of 12.7 J/cm^2^ significantly favored the adhesion of *S. mutans* to primary dental enamel [21]. Moreover, the present focus of research on caries prevention based on Er:YAG lasers has been mainly on the enamel acid resistance, morphological and chemical composition changes, and the effect of combined fluoride and fissure sealing, with less focus on the microbial changes [21–23]. Thus, this study analyzed the structure and diversity of the supragingival plaque microbial community in children with multiple caries using bacterial 16S rRNA sequencing before and 1 month after fluoride treatment alone or combined with Er:YAG laser treatment to provide a feasible reference for the clinical application of the Er:YAG laser and fluoride.

## Materials and methods

### Experimental subject

The study was approved by the Research Ethics Committee of Guangzhou Medical University (No. LCYJ2021042). Written informed consent was obtained from the children and their parents. Children who visited the Paediatric Dentistry Department of Affiliated Stomatology Hospital of Guangzhou Medical University from October to November 2021 were selected. The inclusion criteria for clinical cases were as follows: 1) children with over 8 filled teeth who completed total dental treatment and who did not have untreated caries, pulpitis, periapical inflammation, mucosal diseases; 2) children with at least two primary molars without metal crown repair on each side, and the buccal surfaces of molars were not filled with resin; 3) children with no history of taking antibiotics and who did not use fluoride or undergo supragingival scaling over 3 months; and 4) children and parents who gave informed consent and cooperated with the treatment. The diagnostic criteria used for dental caries were based on the standards of the World Health Organization (WHO) Basic Methods of Oral Health Survey (5th edition) [24]. The exclusion criteria for clinical cases included the following: 1) ongoing orthodontic treatment; 2) presence of systemic diseases (for example, hypertension, diabetes, and others); and 3) a long-term medication history. The final number of samples included was 12 [25, 26].

### Fluoride treatment and Er:YAG laser irradiation process

The surfaces of all teeth were cleaned and dried. The appropriate amount of fluoride varnish (Duraphat, Colgate, USA) was evenly applied on all of the tooth surfaces, and allowed to stand for 1 min. After fluoride coating, the buccal surfaces of the right mandibular primary molars were irradiated by a pulsed Er:YAG laser (Fotona, Erbium-neodymium dual-wavelength laser, Slovenia) under noncontact irradiation 0.5 cm from the target using the R14 handpiece. The set parameters of the Er:YAG laser were 10 Hz, 40 mJ, 50 μs, no water and no gas as recommended by the manufacturer, as shown in Table 1. The laser hand tool was moved around until all the teeth surfaces received one round of irradiation.

**Table 1:** The laser parameters used in this study

Irradiation parameter	Value
Wavelength	2.940 nm
Pulse duration	SSP, 50 μs
Frequency	10 Hz
Energy level	40 mJ
Air/water spray	no water and no gas
Energy density	3.014 J/cm2

### Grouping and supragingival plaque sample collection

The supragingival plaque was sampled as previously described [27, 28]. Parents were informed to ensure that the children did not brush their teeth in the morning and the night before and not to eat or drink water for 2 h before sampling. Before sampling, the children had to gargle with warm water to remove food residues on the tooth surfaces. The sites to be sampled were the buccal surfaces without resin, which were dried beforehand. The buccal surfaces were wiped 3 ~ 5 times to collect as much dental plaque as possible, and then the oral swab with plaque was placed into a sterilization tube. According to whether there was laser irradiation or not and the sampling time, the experimental groups were divided into four groups (*n* = 12/group): supragingival plaque samples of the left mandibular molar before fluoride treatment (CB group); supragingival plaque samples of the right mandibular molar before fluoride+Er:YAG laser treatment (EB group); supragingival plaque samples of the left mandibular molar 1 month after fluoride treatment (CA group); and supragingival plaque samples of the right mandibular molar 1 month after fluoride+Er:YAG laser treatment (EA group). After labeling, all tubes were placed in an insulation box containing dry ice and frozen at − 80 °C for 2 h. A total of 48 dental plaque samples were finally collected and 16S rRNA high-throughput sequencing was performed.

### DNA extraction and sequencing

Dental plaque samples were processed for total DNA extraction, amplification, purification and sequencing. Subsequently, PCR-free libraries based on Illumina Novaseq 6000 (Illumina, San Diego CA, USA) were generated, and paired-end sequencing of the purified DNA was performed. For the raw data, sequence splicing and filtering were performed to obtain high-quality sequences. Then the sequences were clustered at a 97% similarity level using USEARCH (version 10.0) to filter operational taxonomic units (OTUs) with 0.005% of the number of all sequences by default. By using SILVA as the reference database and the naive Bayesian classifier, we obtained the corresponding species classification information for each feature, and then the community composition of each sample was assessed at each level (phylum, class, order, family, genus, and species). The alpha diversity and beta diversity of the samples were assessed using QIIME2 software. Biomarkers with statistically significant differences between groups were identified.

### Statistical analysis

The calculations were performed using SPSS for Windows version 23.0. For normally distributed measurement data, a self-paired t-test was performed for comparison between the pre-and post-treated samples or two samples on the left and right sides. The two groups of data that did not conform to a normal distribution were analyzed using the Mann-Whitney test. For multiple comparisons of data that followed a normal distribution and displayed homogeneity of variance, multiple-group variability was compared using one-way analysis of variance (ANOVA) followed by the Bonferroni post hoc test. The Tamheri method for post hoc testing was used for pairwise comparisons of multiple groups of data with normal distribution but uneven variance. Multiple sets of data that did not conform to a normal distribution were analyzed using the Kruskal-Wallis H test. Statistical differences were considered as those with *P* values < 0.05.

## Results

### Sample characteristics and sequencing data

Twelve children were included in the present study, and 48 plaque samples were collected, including 12 in each group. The age range of children was 4–8 years old, including six boys and girls each. The mean number of decayed, missing, and filled teeth (DMFT/dmft) was 11.75. There were 3,840,942 raw reads, 3,832,915 clean reads, and 3,503,645 effective reads obtained from the 48 plaque samples (Additional file 1: Table S1).

**Fig. 1:**
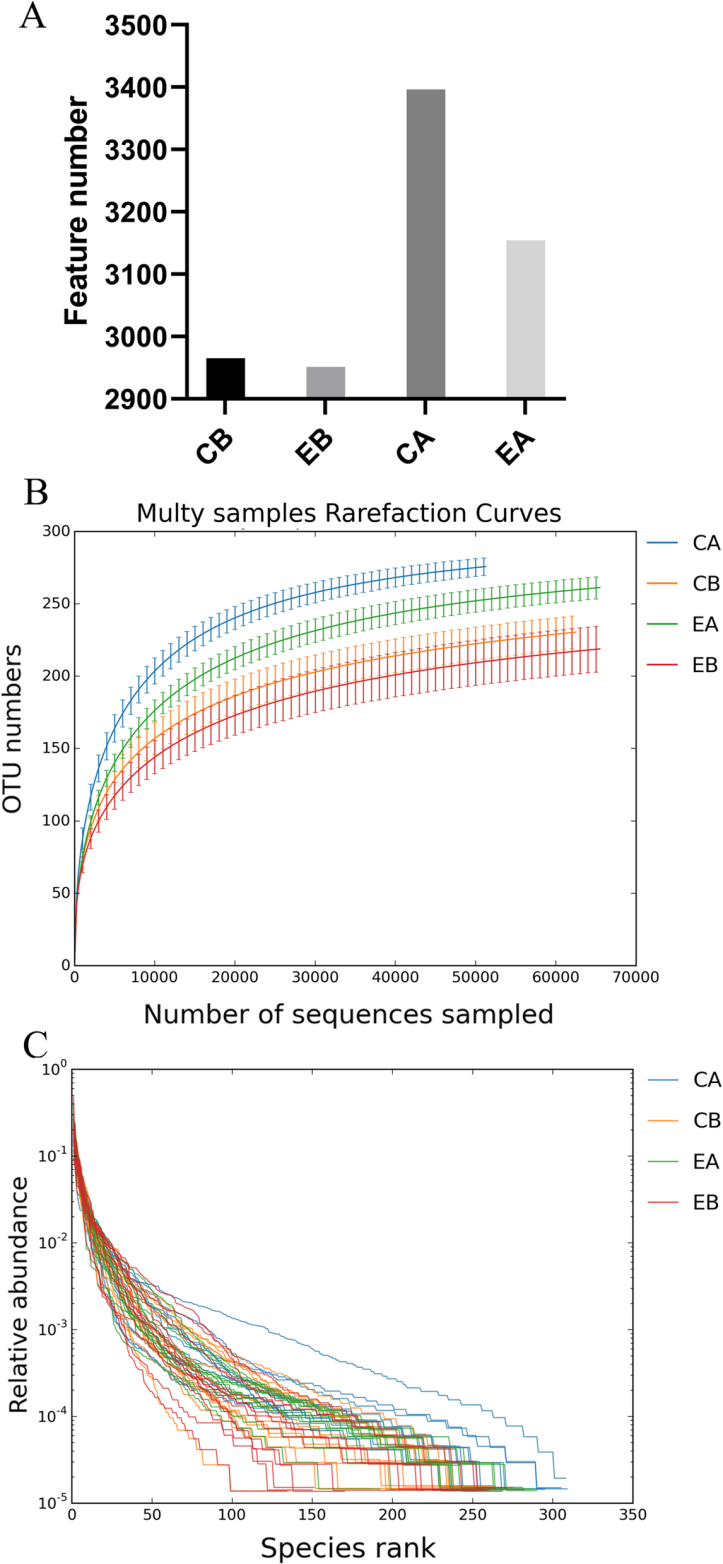
Characteristics of the sequencing data. **A** The analysis of OTUs/ amplicon sequence variants (ASVs). The abscissa is the sample name, and the ordinate is the number of OTUs. Each OTU represents one species; **B** The rarefaction curves of the samples at the 97% similarity level. The abscissa is the number of randomly selected sequencing reads, the ordinate is the number of features obtained based on the number of sequencing reads, and each curve represents one sample, indicated with different colors; **C** The rank abundance curves. Wider abundance curves indicate richer and broader species composition and higher uniformity of species composition

A total of 12,466 OTUs were obtained, including 2965 OTUs in the CB group, 2951 OTUs in the EB group, 3396 OTUs in the CA group, and 3154 OTUs in the EA group (Fig. 1A). The CA group had the highest numbers of OTUs, followed by the EA, CB, and EB groups, suggesting that the numbers of species increased after laser or fluoride treatment. The rarefaction curves plateaued after rising in a certain range (Fig. 1B), indicating that the sequencing volume of samples was sufficient for the following data analysis and enough to reflect the diversity of the microbial community. As shown in Fig. 1C, the richness and uniformity of microbial species in the CA and EA groups were greater than those in the CB and EB groups.

### The microbial diversity changed after laser and fluoride treatment

**Fig. 2:**
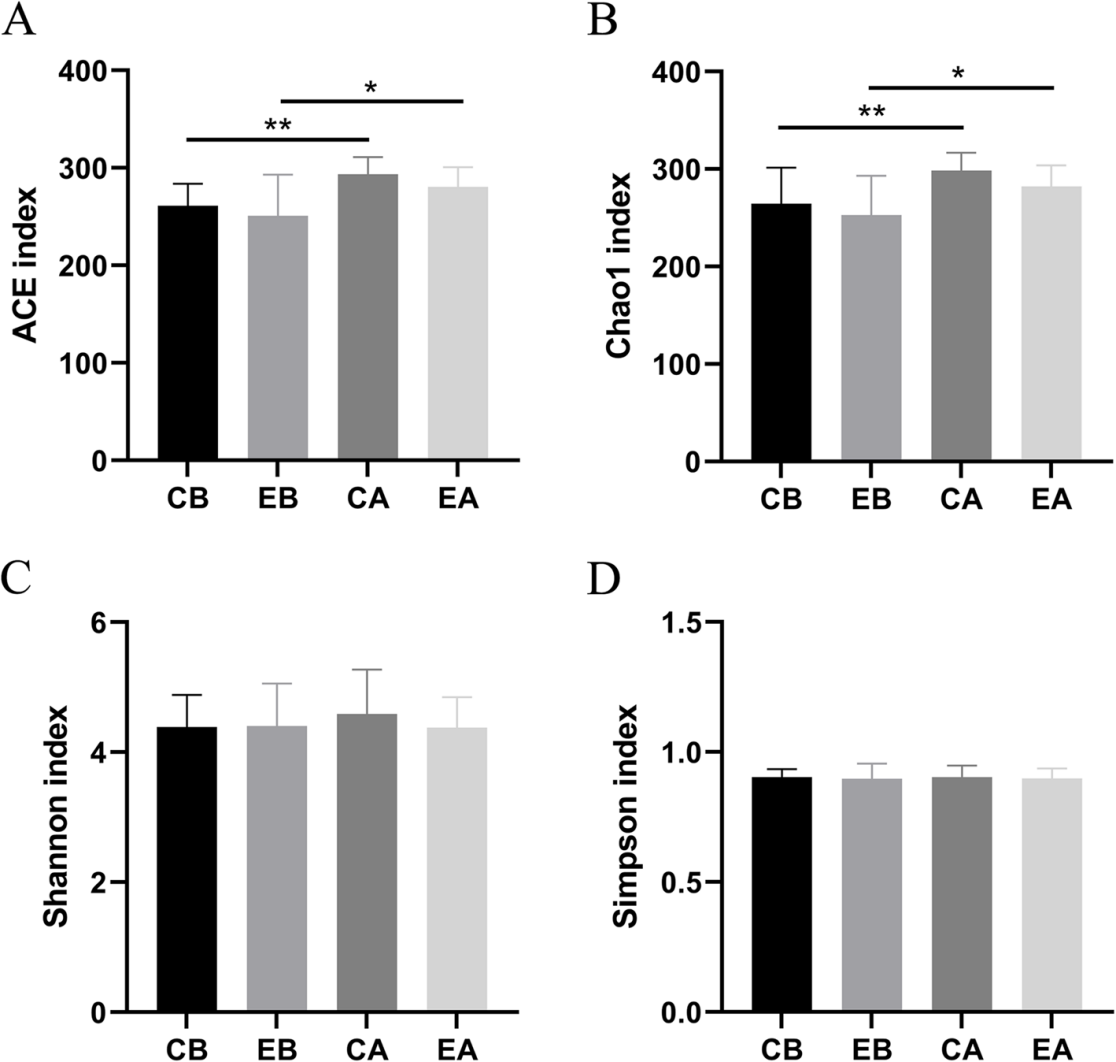
The alpha diversity of the subgingival plaque microbiota. **A** ACE index; **B** Chao1 index; **C** Shannon index; **D** Simpson index; *P < 0.05, **P < 0.01

Regarding the abundance-based coverage estimator (ACE) index (Fig. 2A) and Chao1 index (Fig. 2B), the number of species in the CA group was higher than that in the CB group, and the number of species in the EA group was higher than that in the EB group (*P* < 0.05), while there was no significant difference between the CB group and EB group or the CA group and EA group (*P* > 0.05), illustrating that the baseline microbial abundance was consistent between the two groups before treatment, but after treatment, the microbial abundance in dental plaques increased. Based on the Shannon index (Fig. 2C) and Simpson index (Fig. 2D), no significant difference in microbial species diversity among all groups was observed (*P* > 0.05).

**Fig. 3:**
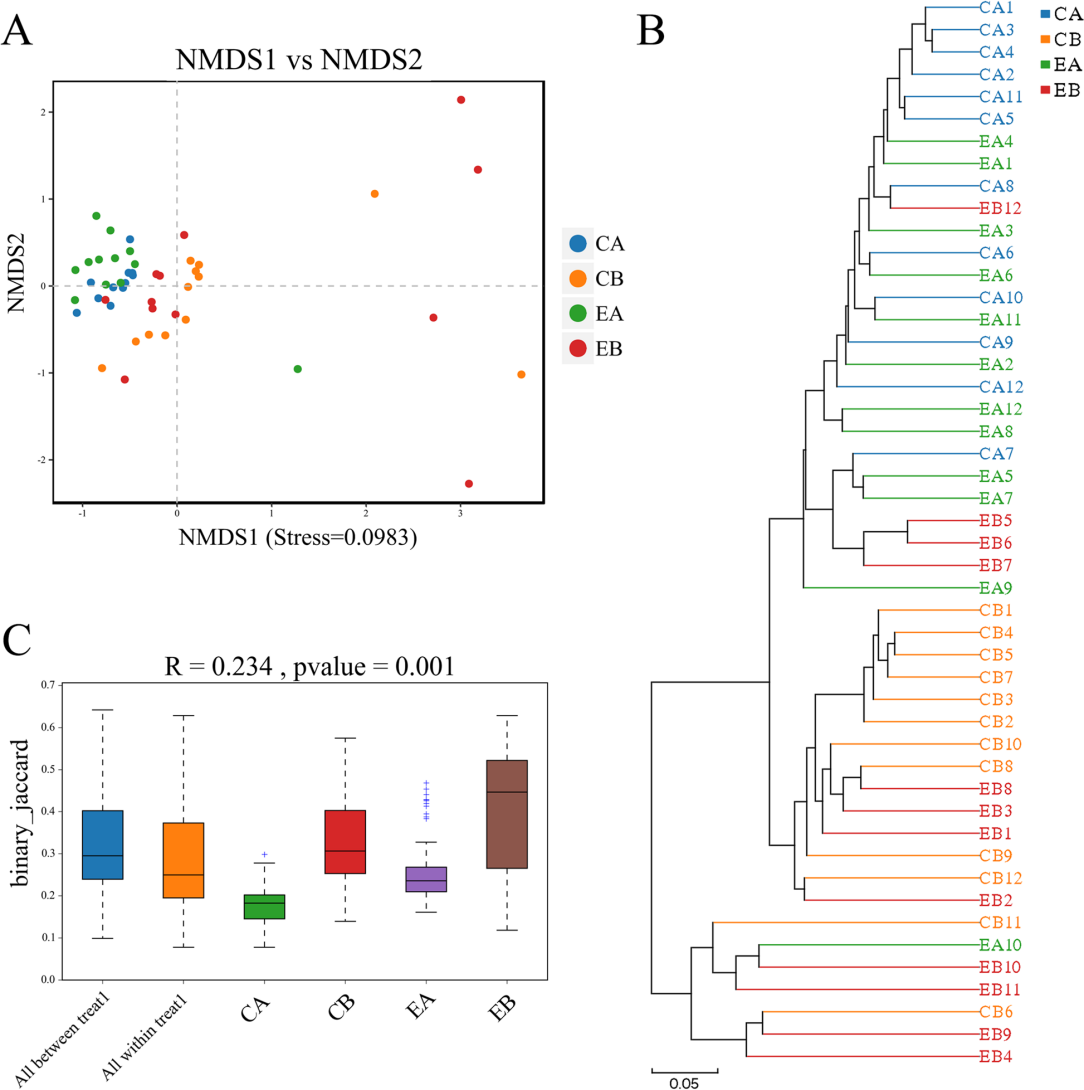
Comparison of beta diversity among supragingival plaque groups. **A** Plot of NMDS analysis of dental plaque samples. Each point represents a different sample. When the stress is less than 0.2, the NMDS analysis has a certain reliability, and the closer the samples are on the coordinate map, the higher the species community similarity; **B** The UPGMA cluster tree of dental plaque samples. Different colours represent different groups. The closer the samples in the sample hierarchy cluster tree, the shorter the branch length, and the more similar the species composition of the two samples; **C** The ANOSIM analysis of dental plaque samples. The ordinate represents the beta distance

As shown in Fig. 3A, which depicts the results of the Non-Metric Multidimensional Scaling NMDS analysis, most of the data points in the CB and EB groups were clustered together, and there were six points scattered on the other side of the graph, but these samples were different from samples obtained from the other groups after treatment. The data points in the CA and EA groups were concentrated. Unweighted Pair Group Method with Arithmetic Mean analysis (UPGMA; Fig. 3B) demonstrated that the microbial community composition was similar between the CB and EB groups or between the CA and EA groups. The microbial community observed after treatment was more similar to that before processing. To assess whether there were significant differences in beta diversity between different samples, an analysis of similarities (ANOSIM; Fig. 3C) was further performed, which demonstrated a significant difference between or within groups (*P* < 0.05).

### No significant differences in the distribution of predominant microorganisms before and after treatment

According to the clustering and annotation of OTUs, the dental plaque microbiota assessed in this study exhibited 16 phyla, 29 classes, 60 orders, 96 families, 167 genera, and 191 species of bacteria. The number of taxa in the 4 groups at each taxonomic level is shown in Table 2.

**Table 2:** Number of species annotations in the four groups at each taxonomic level

Groups	Phylum	Class	Order	Family	Genus	Species
CB	14	26	54	82	136	156
EB	16	29	54	87	147	171
CA	16	29	60	95	163	187
EA	16	29	57	89	146	167

**Fig. 4:**
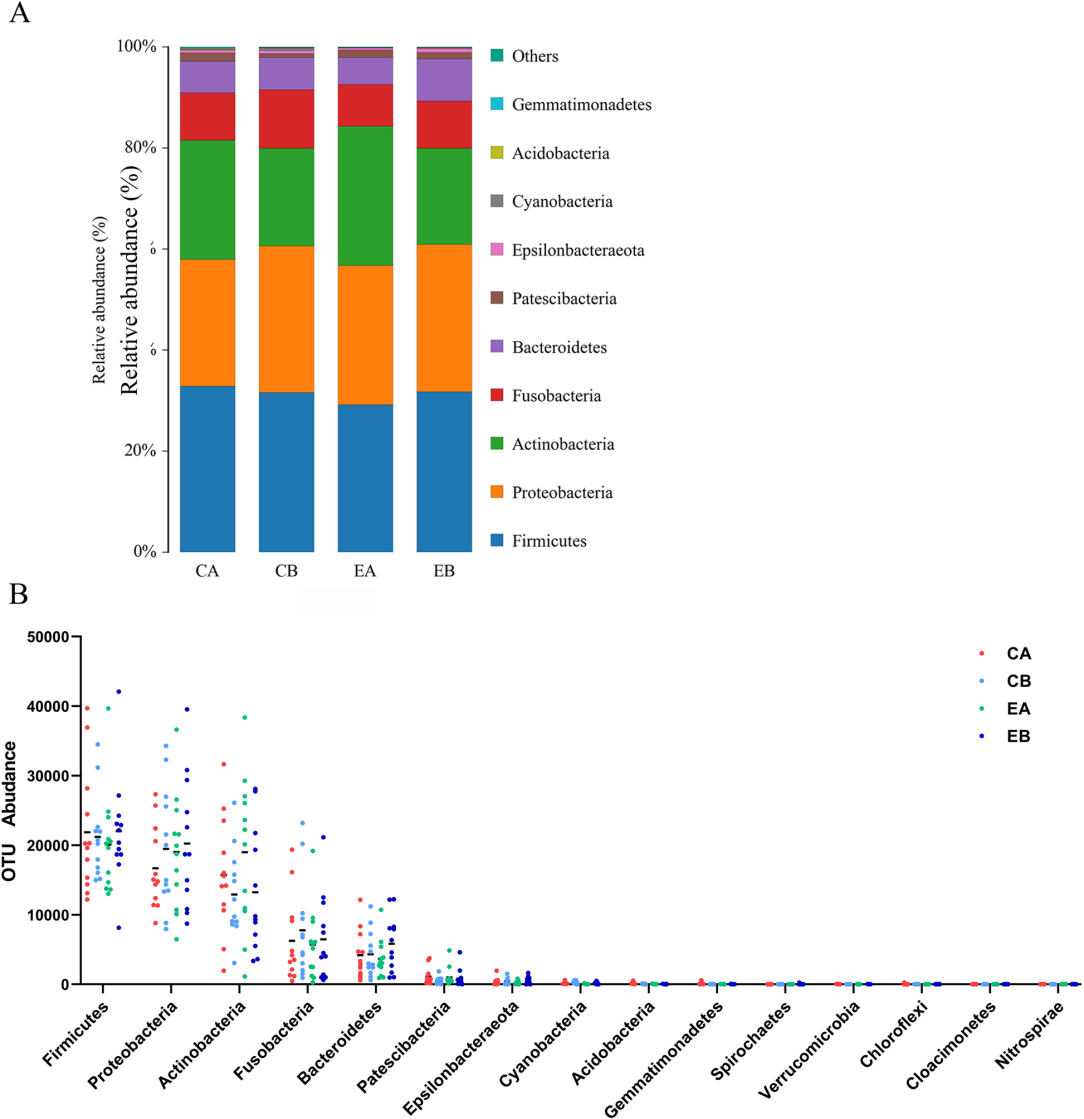
Predominant microbes in the supragingival plaques at the phylum level. **A** The distribution of microbial phylum levels within dental plaques; **B** The abundance of microbiological phyla levels within dental plaques

At the phylum level (Fig. 4, and Additional file 1: Figs. S1, S3), the top 10 dominant phyla in relative abundance were Firmicutes (33.4%), Proteobacteria (22.9%), Actinobacteria (23.9%), Fusobacterium (10.3%), Bacteroides (7.1%), Patescibacteria (1.4%), Epsilonbacteraeota (0.6%), Cyanobacteria (0.3%), Acidobacteria (0.1%), and Gemmatimonadetes (0.1%). Regarding Firmicutes, the EA group (29.2%) had the lowest abundance, followed by the CB (31.6%), EB (31.7%), and CA (32.9%) groups (*P* > 0.05). Regarding Proteobacteria, the relative abundances between CA and EA groups were similar, and lower than those in the CB and EB groups (*P* > 0.05). Regarding Fusobacteria and Bacteroidetes, the abundance was the lowest in the EA group, which was lower than that in the EB group, and the relative abundance in the CA group was lower than that in the CB group, but the difference was not statistically significant (*P* > 0.05). The relative abundance of Actinobacteria did not differ much among the groups (*P* > 0.05).

**Fig. 5:**
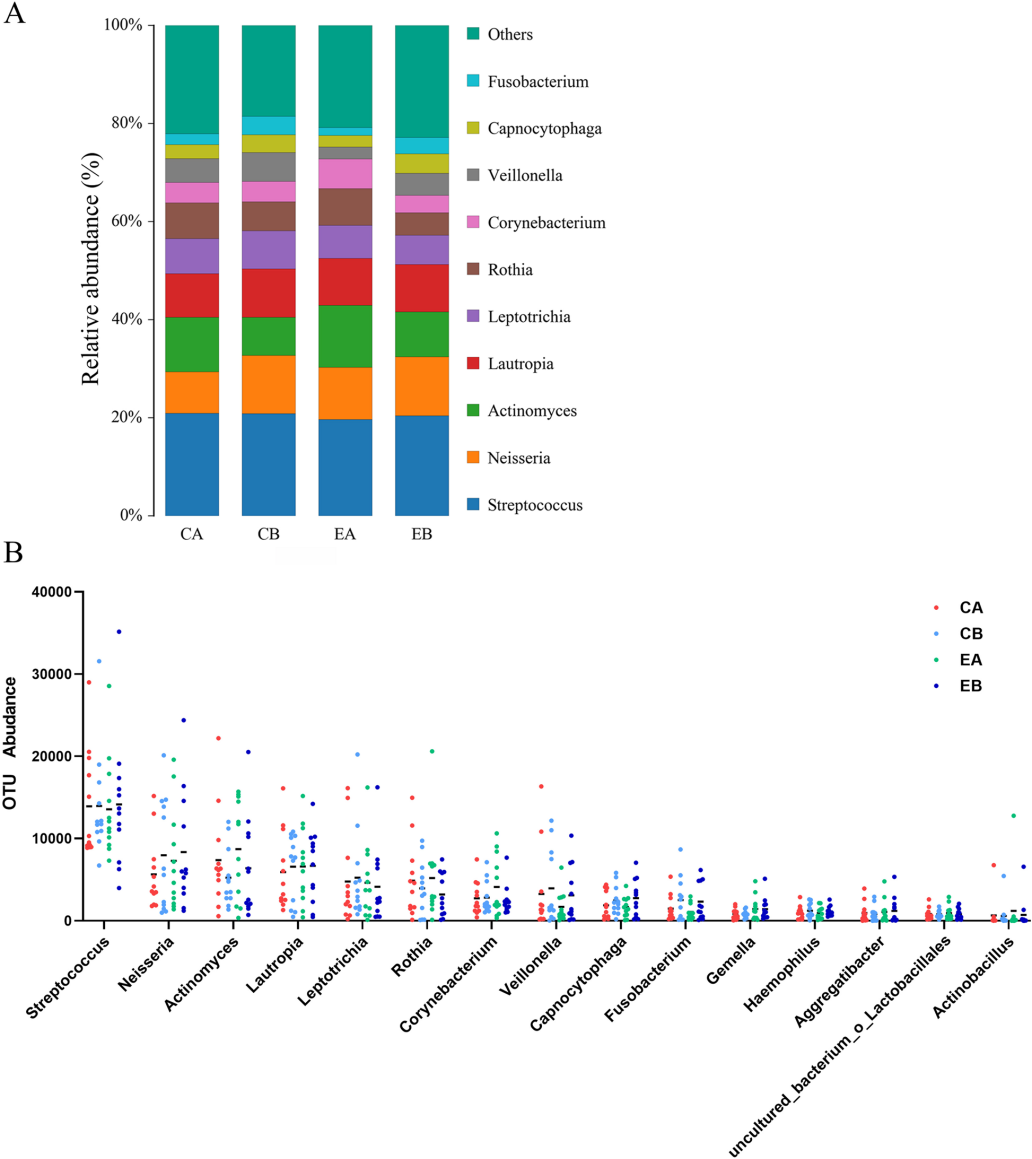
Predominant microbes within supragingival plaques at the genus level. **A** The distribution of microbial genus levels within dental plaques; **B** The abundance of microbiological genus levels within dental plaques

At the genus level (Fig. 5, and Additional file 1: Figs. S2, S4), the top 10 dominant bacterial genera in relative abundance were Streptococcus (20.5%), Neisseria (10.7%), Actinomyces (10.2%), Lautopia (9.5%), Leptotrichia (6.8%), Rothia (6.4%), Corynebacterium (4.5%), Veillonella (4.5%), Capnocytophaga (3.2%), and Fusobacterium (2.7%). The abundance of Actinomyces, Rothia, and Corynebacterium was the highest in the EA group, while the abundance of Streptococcus, Veillonella, and Fusobacterium was the lowest in the EA group, and the abundance among the other groups was similar, and the difference was not statistically significant (*P* > 0.05) .

### Biomarkers of supragingival plaque microbiota among groups

**Fig. 6:**
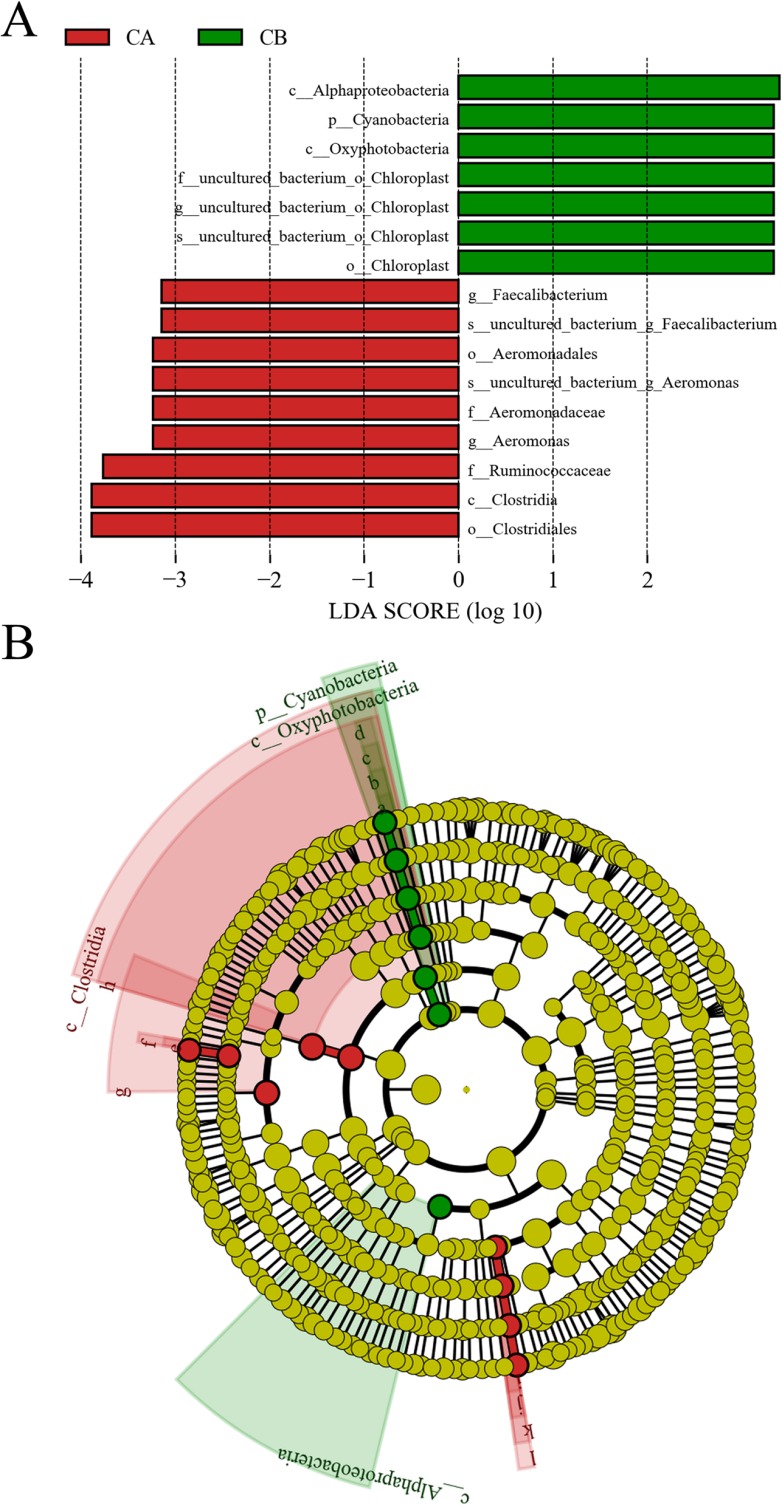
The LEfSe analysis of supragingival plaques among groups. **A** Bar graph of LDA values. The default score was set to 3.0, with significantly different species among groups being considered when the LDA score was greater than this value; **B** Cladogram. The circles radiating from inside to outside represent the classification level from phylum to species. Each node represents a microbe at that level, and the size of the node is proportional to the relative abundance. Sixteen dominant taxa are identified in the diagram. p, phylum; c, class; o, order; f, family; g, genus

As shown in Fig. 6, through comparative analysis for the microorganisms with significant differences and with linear discriminant analysis (LDA) scores above 3.0 based on linear discriminant analysis effect size (LEfSe) analysis, the taxonomic biomarkers in Group CB were Alphaproteobacteria, Cyanobacteria, Oxyphotobacteria, and Chloroplast. The taxonomic biomarkers in Group CA were Clostridia, Clostridiales, Ruminococcaceae, Aeromonadales, Aeromonadaceae, Aeromonas, and Faecalibacterium. No differences in microbial abundance were observed between the EB and EA groups.

In the CA group, some bacterial genera with small relative abundances, such as Faecalibacterium, Vibrio, and Shewanella, exhibited significantly higher abundances than those in the other groups (*P* < 0.05, Additional file 1: Fig. S5). The relative abundance of Faecalibacterium, Vibrio, and Shewanella in the EA group was higher than that in the EB group.

## Discussion

Caries is a multifactorial infectious disease, and its occurrence is mainly mediated by the microecological imbalance caused by acid-producing and acid-resistant microorganisms [29]. The results of 16S rRNA high-throughput sequencing in the present study revealed no significant differences in the predominant microbial composition of the dental surface in terms of supragingival plaques treated with fluoride alone or those treated with fluoride combined with the Er:YAG laser. As is well known, the composition of oral microecology also constantly changes over time. All of the children selected in the present study exhibited a high incidence of caries. Although oral hygiene in children has been improved, the oral environment promotes the predominance of cariogenic bacteria in the long term, and it is difficult to achieve significant changes with short-term treatment. As reported, fluoride affects biofilm formation and reduces acid production in *S. mutans* biofilms, but this antibiofilm activity decreases over time [30]. The period assessed in the present study was 1 month after treatment, and less antibacterial activity due to fluoride was observed at this time. Such findings could partially explain the lack of no significant differences in the dominant bacteria in the short term.

There is still a debate regarding the use of Er:YAG laser irradiation before or after fluoride coating. To achieve better results in enhancing tooth structural resistance and enamel hardness, Valizadeh et al. [31] proposed the application of fluoride varnish or gel before laser irradiation. Er:YAG laser irradiation after the application of fluoride has the potential to melt and seal the dentin tubules, thereby preventing the fluoride from going further into the dentine. Following fluoride application, the use of Er:YAG laser with subablative energy led to greater microhardness, elastic modulus, and fluoride absorption in dentin than the use of laser or fluoride treatment alone [32]. The synergistic effects of Er:YAG laser irradiation and fluoride can mainly be attributed to the thermal efficiency of the laser [33]. On the one hand, laser irradiation promotes the formation of tiny finger-like processes on the enamel surface, and such a structure enables more fluoride to enter the dissolved layer of the enamel surface to form fluorapatite [34, 35]. On the other hand, laser irradiation can lead to stronger binding between fluoride particles on the enamel surface, resulting in reduced enamel solubility [36]. Based on the aforementioned mechanisms, in the present study, a project was designed to illuminate the dental surface with Er:YAG laser after fluoride coating.

The rarefaction curves in the present study plateaued after rising within a certain range, indicating that the sample sequencing volume in the study was sufficient. According to the changes in the alpha diversity and abundance curve, the number of bacterial species increased after fluoride treatment alone or after fluoride treatment in combination with Er:YAG laser treatment. The increased microbial diversity may be associated with altered enamel chemical structure [37]. Such findings are partly consistent with the findings of Widyarman et al. [38], who found that casein phosphopeptide amorphous calcium phosphate fluoride (CPP-ACP/F) treatment regulated the microbial composition of dental plaques, thereby leading to a healthier community that differed from the dysregulated oral microbiota. The occurrence of caries seems to be accompanied by a decline in the diversity of the plaque microbial community. From a microbiological perspective, plaque microbiosis is the cause of childhood caries, which is referred to as the “ecological plaque hypothesis”. As reported, children without caries had a more obvious microbial community diversity than children with caries [39]. The improvement in the microbial community diversity associated with the dental surface is conducive to the balance of oral microbial ecology and reduces the incidence of caries.

In the present study, through species analysis, Firmicutes, Proteobacteria, Actinobacteria, Fusobacteria, and Bacteroides, and especially the first three species, were found to be predominant in the supragingival plaque microbiota. The abundances of Proteobacteria, Fusobacteria and Bacteroides decreased after the use of fluoride alone or in combination with Er:YAG laser treatment, and this decrease was more significant in the fluoride+Er:YAG laser treatment. The decrease in the proportion of the aforementioned cariogenic bacteria, especially Firmicutes, leads to a healthier distribution of oral microflora structure [28], probably due to the local environmental changes caused by Er:YAG laser irradiation. A prospective study by Widyarma involving 10 children with caries receiving weekly topical fluoride (CPP-ACP/F) for 1 month also showed a significant reduction in Bacteroides and Firmicutes abundances and a significant increase in Proteobacteria abundances [38]. Different results were assumed to be related to the frequency of fluoride application, the concentration used, oral health status, dietary habits, the selected study subjects, and the sampling location.

At the genus level, Streptococcus, Neisseria, Actinomyces, and Lautropia were dominant. Overall, Streptococcus had the lowest proportion and abundance in the EA group. When each patient was analyzed, not all patients exhibited a decrease in abundance. Notably, several of the nondominant bacterial genera exhibited different characteristics. The proportions of the Fusobacterium were higher in 8 samples in the CA group than those in the CB group, but lower in 8 samples in the EA group than in the EB group. The proportions of Actinomyces, Corynebacterium, and Rothia were higher in 8 samples in the EA group than those in the CA group, while the proportion of Veillonella was the opposite. Therefore, there were both commonalities and differences in the microbial community of dental surface gingival plaques after the combined treatment of Er:YAG laser and fluoride.

The combination of Er:YAG laser and fluoride seemed to be more detrimental to the survival of Fusobacterium and Veillonella than the use of fluoride alone. Fusobacterium is a strictly anaerobic Gram-negative bacillus, among which only a small number of strains are known to weakly ferment glucose and fructose [40]. Notably, Veillonella is more closely related to the dental pulp or periapical infection [41, 42]. A positive correlation between Veillonella abundance and the occurrence of caries has been indicated [43]. In the present study, a high concentration of fluoride paint was used, which formed a temporary and insoluble CaF^2^-like temporary layer on the enamel surface. The thermal effect of Er:YAG laser could promote the formation of fluorapatite and help form a “fluorine reservoir” locally [35], which may be the reason for the difference in the proportion of bacteria. Based on changes at the phylum level, it was observed that the combination treatment of Er:YAG laser and fluoride could reduce the proportion of cariogenic bacteria, such as Firmicutes, Proteobacteria, and Streptococcus. As such, Er:YAG laser and fluoride combined treatment has a certain value in caries prevention. The present study had some limitations. Plaque microecology is related to various factors such as sucrose intake and oral hygiene, and control variables should be studied in the future. Thus, the sample size can be expanded in the future, and subgroup analyses can be conducted according to different caries risk levels, dentitions, sex, and other factors. Subsequently, dynamic research should be conducted across multiple time points.

## Conclusion

There were no statistically significant differences between the predominant microbial composition before and after the synergistic application of Er:YAG laser and fluoride, but the microflora diversity increased and the proportion of microorganisms changed. The abundance of microorganisms associated with caries occurrence, including Proteobacteria, Fusobacteria, and Bacteroidetes all declined, while the abundance of Faecacterium, Fastidiosipila, Vibrio, and Shewanella increased. Firmicutes, Streptococcus, Fusobacterium, and Veillonella abundances were lower in the laser and fluoride combination treatment groups than in the fluoride treatment group. The combined application of fluoride and Er:YAG laser is conducive to the microecological balance of plaques, in addition to being simple and noninvasive.

## Supplementary Information

**Additional file 1: Table S1.** The basic information and sequence data of the samples. **Figure S1.** Relative abundance of the top 10 taxa in each sample at the phylum level. **Figure S2.** Relative abundance of the top 10 taxa in each sample at the genus level. **Figure S3.** The heat map of microbes relative abundance of the top 16 taxa for 48 samples at the phylum level. The color gradient from blue to red indicates the relative abundance from low to high. **Figure S4.** The heat map of microbes relative abundance of the top 100 taxa for 48 samples at the genus level. The color gradient from blue to red indicates the relative abundance from low to high. **Figure S5.** Expression of bacterial genera with small relative abundance at the genus level.

## Data Availability

The datasets generated and/or analyzed during the current study are not publicly available due to privacy and ethical concerns but are available from the corresponding author on reasonable request.

## Abbreviations

Er:YAG: Erbium:yttrium-aluminium-garnet
FDA: American food and drug administration
OTUs: Operational taxonomic units
ACE: Abundance-based coverage estimator
LDA: Linear discriminant analysis
LEfSe: Linear discriminant analysis effect size
