# Supplementary material for: The impact of Er:YAG laser combined with fluoride treatment on the supragingival plaque microbiome in children with multiple caries: a dynamic study
Source: BMC Oral Health. 2022 Nov 24;22:537. doi: 10.1186/s12903-022-02537-z (PMC9685943; doi:10.1186/s12903-022-02537-z)
Supplement: Supplementary file 1 — Additional file 1: Table S1. The basic information and sequence data of the samples. Figure S1. Relative abundance of the top 10 taxa in each sample at the phylum level. Figure S2. Relative abundance of the top 10 taxa in each sample at the genus level. Figure S3. The heat map of microbes relative abundance of the top 16 taxa for 48 samples at the phylum level. The color gradient from blue to red indicates the relative abundance from low to high. Figure S4. The heat map of microbes relative abundance of the top 100 taxa for 48 samples at the genus level. The color gradient from blue to red indicates the relative abundance from low to high. Figure S5. Expression of bacterial genera with small relative abundance at the genus level. [file 12903_2022_2537_MOESM1_ESM.doc]

**Supplementary Information**

Additional file 1: Table S1 The basic information and sequence data of the samples.

| Sample  ID | Gender | Age  (Year) | DMFT/dmft | Raw reads num | Clean reads num | Effective reads num |
| --- | --- | --- | --- | --- | --- | --- |
| CB1 | Female | 8 | 9 | 79877 | 79700 | 71933 |
| CB2 | Male | 5 | 8 | 79851 | 79695 | 71467 |
| CB3 | Female | 5 | 13 | 79944 | 79798 | 74431 |
| CB4 | Male | 6 | 8 | 80072 | 79902 | 70220 |
| CB5 | Female | 5 | 15 | 79790 | 79633 | 72678 |
| CB6 | Female | 4 | 11 | 79907 | 79698 | 71286 |
| CB7 | Female | 8 | 9 | 80131 | 79932 | 71674 |
| CB8 | Male | 5 | 13 | 79935 | 79755 | 75628 |
| CB9 | Male | 5 | 8 | 79769 | 79629 | 69964 |
| CB10 | Male | 4 | 19 | 80223 | 80044 | 75870 |
| CB11 | Male | 8 | 14 | 79944 | 79787 | 73748 |
| CB12 | Female | 7 | 14 | 80306 | 80133 | 65876 |
| EB1 | Female | 8 | 9 | 80423 | 80278 | 72357 |
| EB2 | Male | 5 | 8 | 80436 | 80267 | 75765 |
| EB3 | Female | 5 | 13 | 79811 | 79617 | 74932 |
| EB4 | Male | 6 | 8 | 79704 | 79539 | 71790 |
| EB5 | Female | 5 | 15 | 79971 | 79811 | 73759 |
| EB6 | Female | 4 | 11 | 79877 | 79731 | 73941 |
| EB7 | Female | 8 | 9 | 80542 | 80383 | 72043 |
| EB8 | Male | 5 | 13 | 79965 | 79779 | 74202 |
| EB9 | Male | 5 | 8 | 79989 | 79828 | 70373 |
| EB10 | Male | 4 | 19 | 79965 | 79825 | 74462 |
| EB11 | Male | 8 | 14 | 80108 | 79957 | 73736 |
| EB12 | Female | 7 | 14 | 80046 | 79913 | 68401 |
| CA1 | Female | 8 | 9 | 80155 | 79943 | 73345 |
| CA2 | Male | 5 | 8 | 80075 | 79907 | 72127 |
| CA3 | Female | 5 | 13 | 79937 | 79779 | 73310 |
| CA4 | Male | 6 | 8 | 80094 | 79921 | 76588 |
| CA5 | Female | 5 | 15 | 80194 | 80001 | 74011 |
| CA6 | Female | 4 | 11 | 79659 | 79492 | 73729 |
| CA7 | Female | 8 | 9 | 79910 | 79716 | 74554 |
| CA8 | Male | 5 | 13 | 79788 | 79638 | 74541 |
| CA9 | Male | 5 | 8 | 79945 | 79786 | 73195 |
| CA10 | Male | 4 | 19 | 80291 | 80124 | 75783 |
| CA11 | Male | 8 | 14 | 79871 | 79691 | 71895 |
| CA12 | Female | 7 | 14 | 80189 | 80005 | 67954 |
| EA1 | Female | 8 | 9 | 79909 | 79726 | 73392 |
| EA2 | Male | 5 | 8 | 80167 | 80033 | 72803 |
| EA3 | Female | 5 | 13 | 79949 | 79787 | 73092 |
| EA4 | Male | 6 | 8 | 80005 | 79846 | 75707 |
| EA5 | Female | 5 | 15 | 80289 | 80106 | 73776 |
| EA6 | Female | 4 | 11 | 79845 | 79685 | 72562 |
| EA7 | Female | 8 | 9 | 79882 | 79722 | 72047 |
| EA8 | Male | 5 | 13 | 80175 | 80005 | 75577 |
| EA9 | Male | 5 | 8 | 79742 | 79586 | 71885 |
| EA10 | Male | 4 | 19 | 79693 | 79537 | 75202 |
| EA11 | Male | 8 | 14 | 80202 | 80028 | 72680 |
| EA12 | Female | 7 | 14 | 80390 | 80217 | 73354 |

**
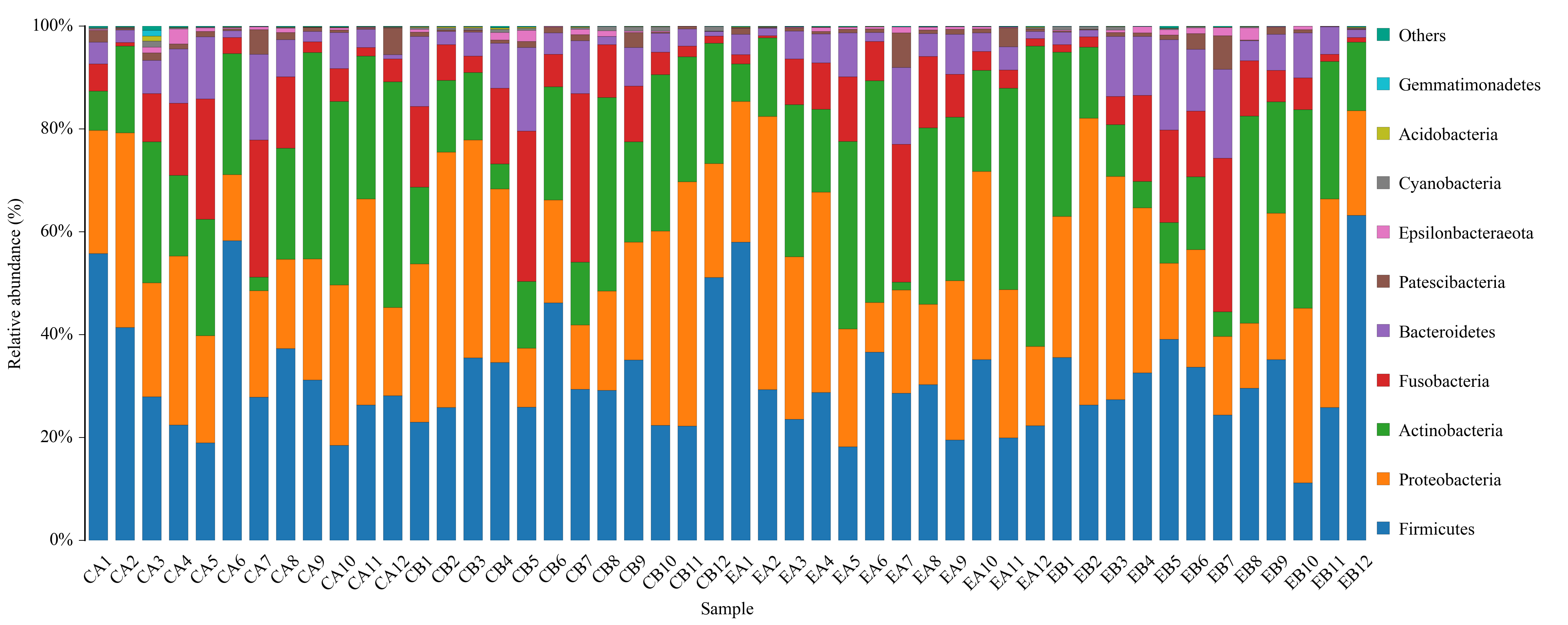
**

**Figure S1 Relative abundance of the top 10 taxa in each sample at the phylum level.**

**
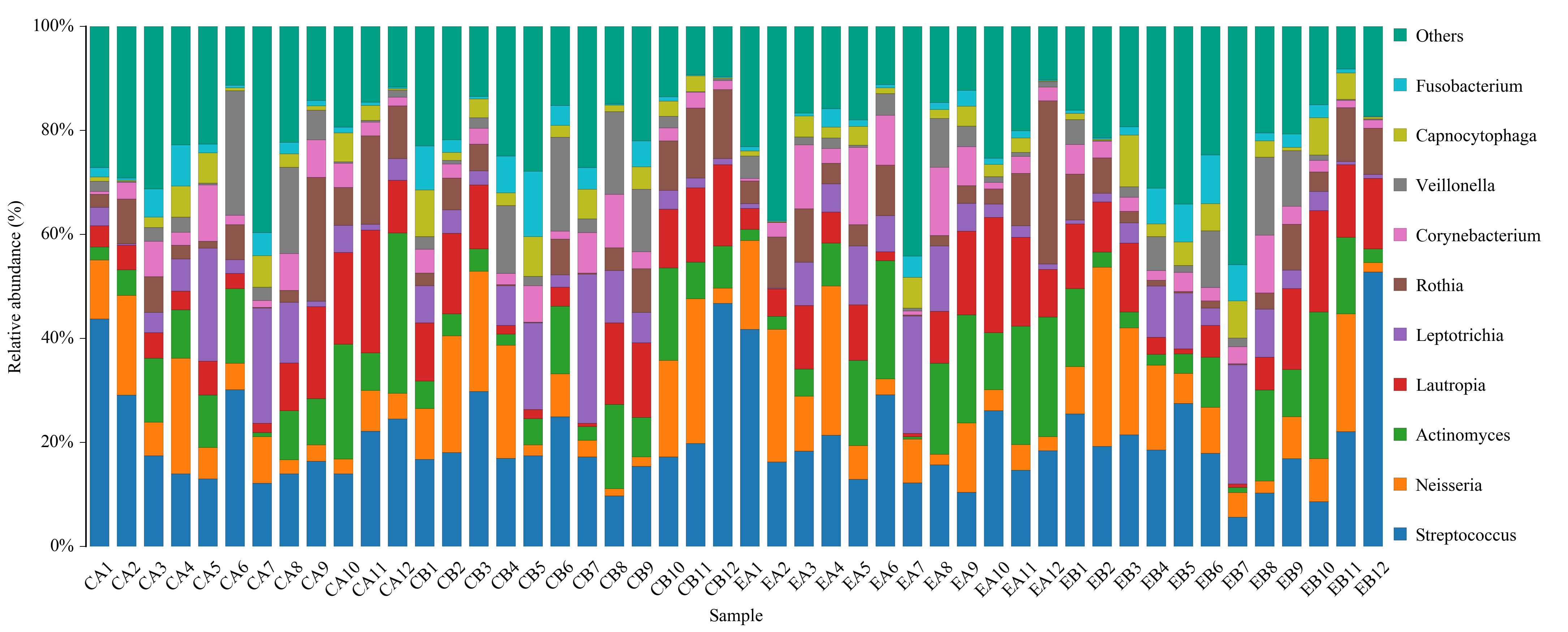
**

**Figure S2 Relative abundance of the top 10 taxa in each sample at the genus level.**

**
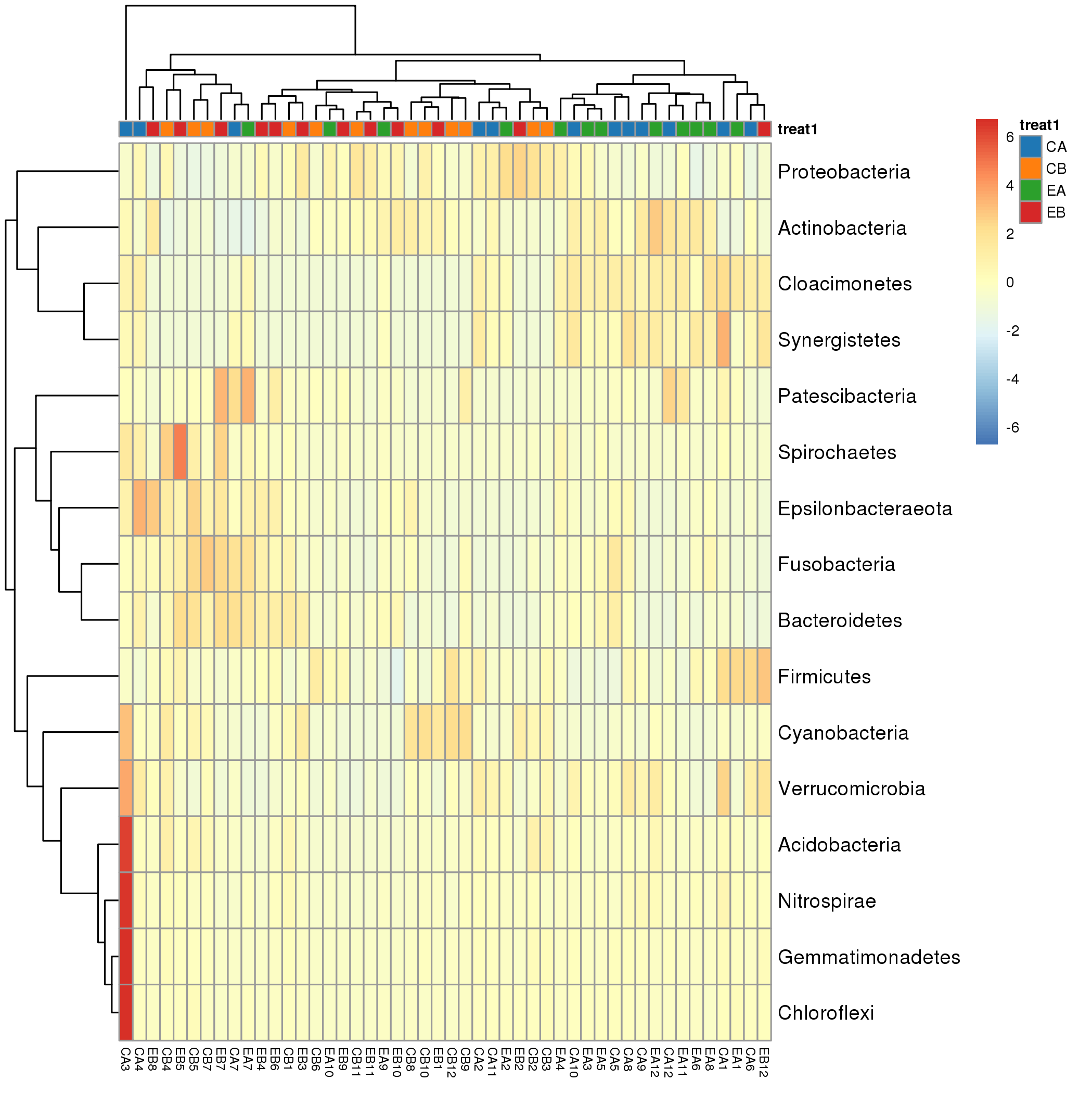
**

**Figure S3 The heat map of microbes relative abundance of the top 16 taxa for 48 samples at the phylum level.** The color gradient from blue to red indicates the relative abundance from low to high.

**
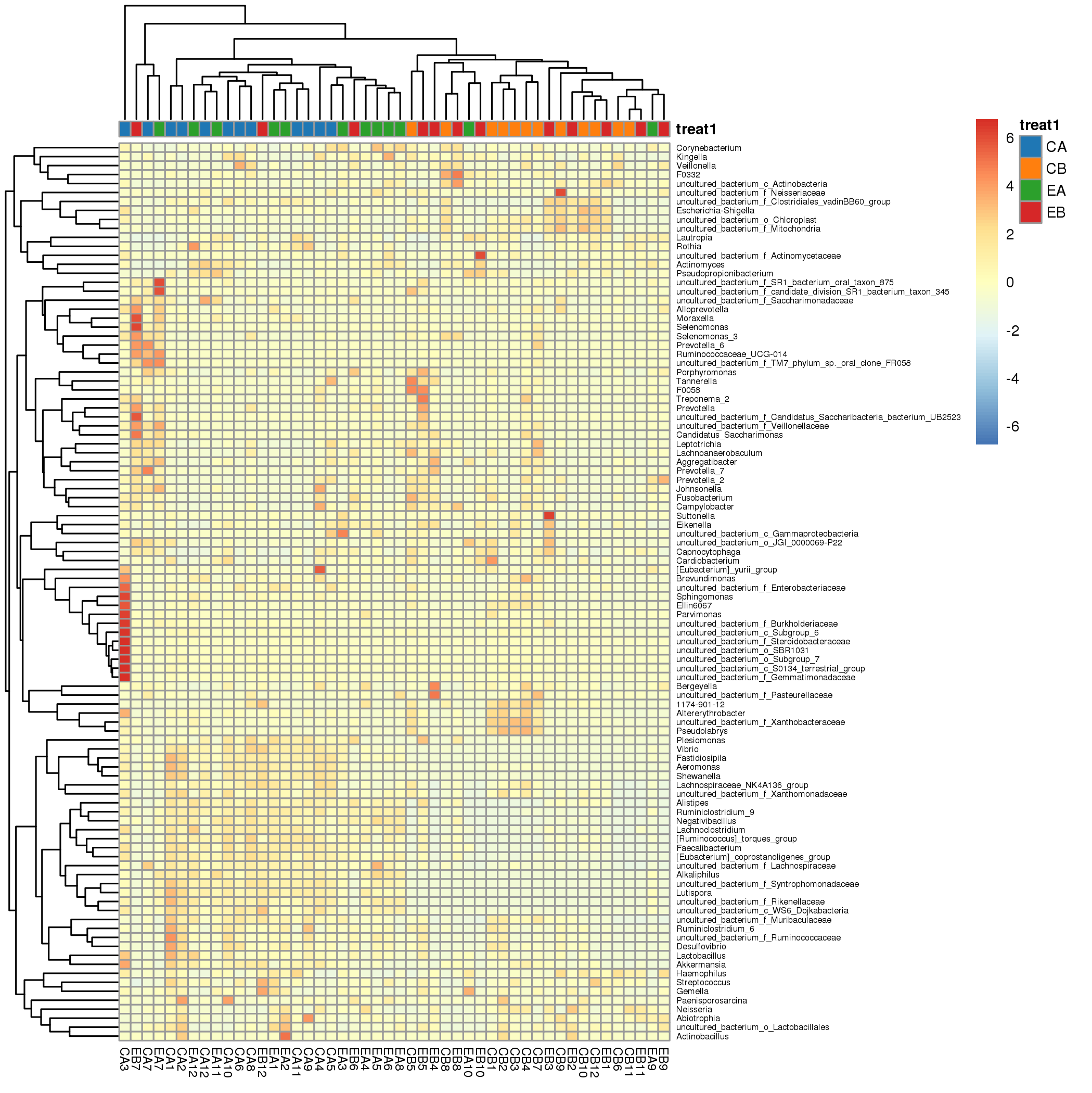
**

**Figure S4 The heat map of microbes relative abundance of the top 100 taxa for 48 samples at the genus level.** The color gradient from blue to red indicates the relative abundance from low to high.

**

**

**Figure S5 Expression of bacterial genera with small relative abundance at the genus level.**
